# Standardizing the lateral fluid percussion model of trauma in marmosets (*Callithrix jacchus*): regional vulnerability and inflammatory response

**DOI:** 10.1590/1414-431X2026e15289

**Published:** 2026-04-27

**Authors:** V. Sanabria, C. Gimenes, S. Romariz, M. Braga, A.S. Gois, M.L. Foresti, L.E. Mello, B.M. Longo

**Affiliations:** 1 Laboratório de Neurofisiologia, Departamento de Fisiologia, Universidade Federal de São Paulo, São Paulo, SP, Brasil; 2 Laboratório de Neurobiologia, Departamento de Fisiologia, Universidade Federal de São Paulo, São Paulo, SP, Brasil; 3 Instituto D'Or de Pesquisa e Ensino, São Paulo, SP, Brasil

**Keywords:** Marmosets, Degeneration, Trauma, Inflammation, Hematomas

## Abstract

Traumatic brain injury (TBI) is a major global health concern. The lateral fluid percussion injury (LFPI) model, widely used in rodents to simulate nonpenetrating TBI, has limited translational applicability due to anatomical differences between rodent and human brains. The common marmoset (*Callithrix jacchus*), a New World primate with a quasi-gyrencephalic brain, offers a promising alternative. This study aimed to standardize the LFPI model in marmosets by comparing trauma responses across parietal and temporal lobes. Ten adult marmosets underwent LFPI in these regions. Lesion volume was measured using Nissl staining; astrocytic and microglial responses were assessed via GFAP and Iba-1 immunofluorescence, and degenerating neurons were identified with Fluoro-Jade B. Righting reflex time and hemorrhage presence were evaluated as injury markers. Percussion aiming at the temporal lobe injury resulted in the most prominent lesions, epidural and subdural hematomas, and significant neuronal degeneration. Astrocytes showed longer processes after temporal trauma in the cortex and fewer branches in the hippocampal region CA1 than in the naive group. In contrast, hippocampal microglia showed fewer elongated branches in CA1 and dentate gyrus (DG), indicative of a reactive phenotype. Our results highlighted region-specific vulnerability, with temporal injury triggering the most pronounced inflammatory and degenerative responses. The marmoset LFPI model effectively mirrored key aspects of human TBI, supporting its translational relevance.

## Introduction

Traumatic brain injury (TBI) is a major global health issue, often described as a silent epidemic due to its significant impact on disability, morbidity, and mortality (1). Preclinical research relies heavily on rodent models that replicate key trauma characteristics such as increased cortical excitability and seizure susceptibility (1,2). However, the smooth (lissencephalic) structure of rodent brains limits their ability to model complex folding and injury patterns of human (gyrencephalic) brains, reducing their translational relevance (3-5).

Non-human primates (NHPs) offer a more physiologically relevant alternative due to their anatomical and genetic similarities to humans (6). Among them, the common marmoset (*Callithrix jacchus*) presents several advantages: small size, high reproductive rate, and strong immunological compatibility with humans (7).

The lateral fluid percussion injury (LFPI) model is a well-established method for inducing nonpenetrating TBI (8). Given their brain structure, marmosets may better replicate cortical injury mechanisms and neurorecovery processes, helping bridge the gap between rodent and primate models in translational neuroscience (7). This study aimed to standardize the LFPI-TBI model in marmosets by identifying the optimal cortical region for trauma induction and assessing region-specific inflammatory and degenerative responses.

## Material and Methods

### Subjects and housing

All procedures were conducted in accordance with Animal Research: Reporting of In Vivo Experiments (ARRIVE), National Institutes of Health (NIH), and IBAMA (Brazilian Institute of Environment and Renewable Natural Resources) guidelines, with approval from the institutional Animal Care and Use Committee (protocol #5138271222). Ten adult marmosets (*C. jacchus*) (3 males, 7 females; age: 2-8 years; weight: 180-360 g) were obtained from the Centro de Manejo e Conservação de Animais (CeMaCAS) and transported to the primate facility of the Federal University of São Paulo. They were housed individually in wire cages (50×50×50 cm) under controlled conditions but within sight and sound of other animals. Additionally, animals were maintained at a stable room temperature (25±2°C), 45-55% humidity, and a 12-h light/dark cycle, starting at 7:00 AM. Animal cages were cleaned and changed twice weekly and assigned randomly to the experimental groups. Moreover, animals received enrichment (branches, wooden swings), water *ad libitum*, and fresh fruits twice daily.

### Groups

Animals were randomly assigned to experimental groups: Naive (no trauma or surgery, n=3; 2 females and 1 male), TBI-Parietal (LFPI to the parietal lobe, n=3; 2 females and 1 male), and TBI-Temporal (LFPI to the temporal lobe, n=4; 3 females and 1 male). Group sizes were limited in compliance with ethical guidelines for NHP research.

### LFPI model of brain trauma

Animals were anesthetized with isoflurane (3% for inhalation and 2% for maintenance, at a flow rate of 1-1.5 L/min in freely breathing oxygen) (SomnoSuite, Kent Scientific, USA), followed by an intradermal injection of lidocaine (10 mg/kg) for local anesthesia. A 15-mm midline incision was made to expose the skull, and a 5 mm burr hole was drilled using an electric drill (Stoelting, USA), ensuring the integrity of the dura mater in all trauma regions. At the craniotomy site, a cannula (a female Luer lock made from a pipette tip cut at the end, forming a cannula with an aperture measuring 5 mm) was fixed with dental cement. After the cannula was securely placed, it was filled with sterile 0.9% saline solution. The female Luer lock on the animal's skull was then connected to the male Luer lock of the fluid percussion device (Model FP301 Signal Conditioner, AmScien Instruments, USA).

**Figure 1:**
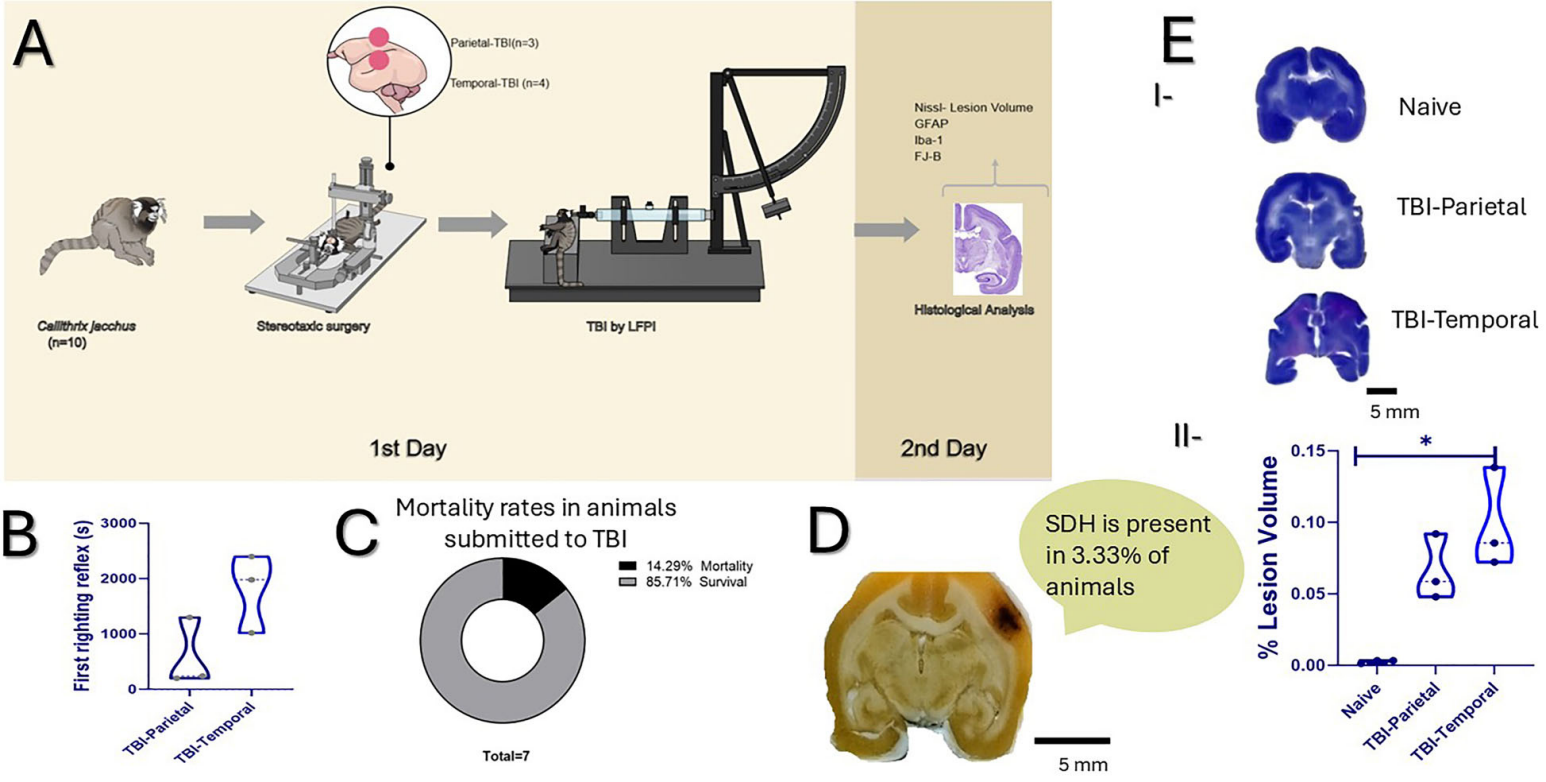
Experimental design and lateral fluid percussion injury (LFPI) model standardization. **A**, Overview of the experimental design. Stereotaxic surgery and induction of traumatic brain injury (TBI) by lateral fluid percussion injury (LFPI) were performed on day 1 in *C. jacchus* (n=7), with three additional animals serving as Naive controls for a total N=10. Twenty-four hours after trauma, animals were euthanized and perfused for histological analyses. **B**, Comparative graph of the first righting reflex time in seconds (s) after injury in TBI-Parietal and TBI-Temporal. **C**, Percentage of mortality among animals subjected to TBI (n=7). **D**, Representative image of a subdural hematoma (SDH) and the percentage of animals presenting SDH following TBI. Scale bars=5 mm. **E**-**I**, Representative Nissl-stained sections illustrating different injury regions and **E**-**II**, lesion volume graph. Data are reported as means±SD. *P<0.05; Mann-Whitney test; n=9 (three animals per group); each dot represents one animal. Scale bars=5 mm.

Stereotaxic coordinates for trauma induction were: parietal (+1.78 mm anterior-posterior (AP) to bregma, +6 mm medial-lateral (ML), +16 mm dorso-ventral (DV)) and temporal (+1.78 mm AP to bregma, +10 mm ML, +13 mm DV). In the temporal region, an additional intramuscular injection of lidocaine (10 mg/kg, *im*) was administered for local anesthesia of the temporalis muscle. The muscle was gently retracted with a scalpel to expose the stereotaxic target area. LFPI was applied at a 17° angle with a 10-15 ms fluid pulse impacting the exposed dura. Pulse pressures were measured by an extracranial transducer and recorded on a storage oscilloscope. The fluid pressure used to induce trauma in this study ranged from 1.38 to 2.07 atm (means±SD): 1.56±0.26 atm; coefficient of variation: 18% (Figure 1A).

The interval between the initiation of isoflurane anesthesia and trauma induction was approximately 25-30 min. Isoflurane was used exclusively for the stereotaxic surgical procedure and was discontinued before injury induction. During stereotaxic surgery, animals were placed on a thermostatically controlled heating blanket to maintain normothermia (36-38°C), with rectal temperature monitored via a probe connected to a heating pad (RightTemp Jr., Kent Scientific, USA). Physiological parameters were continuously monitored, including heart rate (230-400 bpm), respiratory rate (∼100 breaths/min), and oxygen saturation, using a pulse oximeter with paw sensors (Kent Scientific). Mucous membrane color was also assessed to ensure stable physiological conditions. Heart rate and respiratory rate were recorded using a pulse oximeter and a respiratory monitoring sensor. At the time of injury induction, animals were fully recovered from isoflurane anesthesia. Following trauma, the cannula was removed, and the incision was closed using 2-0 non-absorbable nylon sutures. Subsequently, recovery was assessed by measuring the righting reflex, defined as the time required to return to a bipedal position. Animals received post-surgical care, including pentabiotic (0.1 mL/kg, *im*, Zoetis Brasil, Brazil) and flunixin meglumine (1 mg/kg, *ip*, Brazil). They were kept on a heated blanket and hydrated until they had fully recovered. At 24 h post-trauma, animals were deeply anesthetized with pentobarbital (100 mg/kg, *ip*) and transcardially perfused with phosphate-buffered saline followed by 4% formaldehyde.

### Brain tissue processing

The brains were removed from the cranium, examined for hematomas, and post-fixed in 4% formaldehyde for 24 h. Brains were then cryoprotected in 30% sucrose until they showed signs of dehydration, usually in two weeks. Immediately after this period, the brains were dried and frozen at -80°C. Subsequently, the brains were sectioned in a cryostat (Leica CM1850, Germany) in coronal brain sections (40-µm thick). The sections were then stored in an anti-freezing solution (300 g of sucrose, 500 mL of PBS solution, and 300 mL of ethylene glycol) at -20°C until further histological and immunohistochemical analysis.

### Histological Nissl staining

Brain sections were stained with cresyl violet (Nissl) to evaluate lesion volume. Brain slices were selected from the anti-freezing solution and washed five times in 0.1 M PBS solution (phosphate buffer solution; 5.52 g of monobasic sodium phosphate plus 21.88 g of dibasic sodium phosphate), then mounted on gelatin-coated glass slides and stained for 5 min with 1% cresyl violet dissolved in distilled water and filtered. Stained slides were dehydrated for 1 min in 100, 96, and 70% ethanol, cleared in xylene for 2 min, covered with Entellan^®^ mounting medium (Merck, Germany), and coverslipped. Lesion areas were quantified in ImageJ (NIH, USA) as the mean of 3-5 slides per animal, using the formula (9): Lesion volume = volume of contralateral hemisphere - volume of ipsilaterial hemisphere / volume of contralateral hemisphere × 100.

### Immunofluorescence

Immunofluorescence was performed to identify microglia, astrocytes, and their respective nuclei. Free-floating sections previously stored in antifreeze solution were washed five times with 0.1 M PBS to remove any residual antifreeze. The sections were then incubated in a blocking solution consisting of 90 mL of 0.1 M PBS (99.3% of the final composition), 250 µL of Triton X-100 (0.28%), and 400 µL of bovine serum albumin (0.44%) for 30 min at room temperature under constant stirring. After blocking, cells were incubated for 2 h with the following primary antibodies: monoclonal mouse anti-glial fibrillary acidic protein (GFAP) antibody (1:1000, Sigma, G3893, USA) for astrocytes, and a polyclonal primary antibody anti-Iba1 made in rabbit (1:1000, Wako, 019-19741, Norway) diluted in the blocking solution. After washing in PBS, sections were incubated for 2 h with Alexa Fluor™ 568-conjugated goat polyclonal secondary antibodies (1:600; catalog number A11011 for Iba-1 and A11004 for GFAP; Invitrogen, USA). Nuclei were stained with DAPI (4',6-diamidino-2-phenylindole, 1:10.000; Thermo Fisher Scientific, USA). The slides were coverslipped with mounting medium DPX (catalog number # 06522, Sigma-Aldrich, USA).

The slides were analyzed in a blind manner by two investigators, and images were acquired within a week of staining using a 20× objective using a computer-based digitizing image system (Zeiss Axicam 503; Carl Zeiss, Germany) connected to an LSM 810 Confocal Laser Scanning System. The laser and detector were maintained at constant settings during the acquisition of each staining set.

Quantification was performed using integral density and skeleton (number of branches and average branch length) according to the methodology of Marques et al. (10) in Fiji (ImageJ, NIH, http://imagej.nih.gov/ij). Only the ipsilateral side of the cortex (PaC; parietal cortex, CIn; cingular cortex; TE; temporal cortex) and hippocampus regions (CA1, CA3, and dentate gyrus (DG)) were analyzed. For each brain region analyzed (cortex, CA1, CA3, and DG), three images were acquired per region, and quantitative values were averaged across 3 brain sections per animal.

### Fluoro-Jade^®^ B

Fluoro-Jade^®^ B (FJ-B, catalog number # AG310, Merck-Millipore, USA) was used to stain degenerating neurons. Free-floating sections previously stored in antifreeze solution were washed five times with 0.1 M PBS to remove any residual antifreeze. Brain sections were mounted on glass slides, and FJ-B staining was performed as follows: the slides were allowed to dry at room temperature for 5 days. Then, the slices were placed in an oven at 37°C for 20 min.

The slices were then immersed in 100% ethanol for 3 min, followed by 1 min in 70% ethanol and 1 min in distilled water. The slides were then incubated in 0.06% potassium permanganate (KmnO_4_) in distilled water for 15 min. Next, the slides were rinsed in distilled water and transferred to a 0.0001% FJ-B staining solution in 0.1% acetic acid for 30 min. The FJ-B working solution was prepared from a stock FJ-B solution (0.01% in distilled water). After rinsing, the slides were coverslipped with mounting medium DPX (catalog number # 06522, Sigma- Aldrich).

The slides were analyzed in a blinded manner by two investigators, and images were acquired within a week of staining using a 20× objective with a computer-based digitizing image system (Zeiss Axicam 503; Carl Zeiss) connected to an LSM 810 Confocal Laser Scanning System. FJ-B-positive cells were manually counted in ImageJ, employing the multi-point tool for cell counting in the areas of interest of the cortex (PaC, CIn, and TE) and the hippocampus (CA1, CA3, and DG). The borders of the regions of interest (ROIs) were defined as follows: in ImageJ, a rectangular area was drawn on the image, added to the ROI Manager, and saved for consistent use across all images. Only cells fully contained within the ROI were counted; cells located outside or partially crossing the ROI border were excluded from the analysis. For each brain region analyzed (cortex, CA1, CA3, and DG), three images were acquired per region, and quantitative values were averaged across 3 slides per animal.

### Statistical analysis

Data were analyzed using GraphPad Prism (version 8.0 GraphPad Software Inc., USA, http://www.graphpad.com). Prior to statistical analysis, data normality was assessed using the Shapiro-Wilk test, and outliers were identified using the ROUT method (Q=1%). As data did not meet parametric assumptions, non-parametric Mann-Whitney or Kruskal-Wallis test was applied to compare medians across groups, followed by Dunn's *post hoc* test for multiple pairwise comparisons. Data are reported as means±SD, with statistical significance set at P<0.05.

The final sample size (n=10) was determined by the availability of animals provided by a primate conservation center. Due to ethical and logistical constraints, it was not possible to predefine group sizes. A *post hoc* power analysis conducted using G*Power 3.1 (α=0.05) indicated a statistical power of 0.11, reflecting the limitations imposed by the small sample size.

## Results

In our study, the righting recovery average time for both groups was 1190±896 s (∼20 min). When analyzed separately, animals from the TBI-Parietal group recovered in 580±624 s (∼10 min), whereas those from the TBI-Temporal group required 1800±707 s (∼30 min). Despite the apparent difference in recovery times, no statistically significant difference was observed between groups (Mann-Whitney test: U=1, n=6 [3 animals per group], P=0.200) (Figure 1B).

One animal from the TBI-Temporal group died at 1.73 atm (1/7; n=7; Naive group (n=3) was intact) (Figure 1C), and its data were excluded from righting reflex, histological, immunofluorescence, and neurodegeneration analyses. Upon brain dissection, epidural hematomas (EDH) were found in 33% of animals with parietal TBI (1/3) and in all animals with temporal TBI (3/3). Moreover, two temporal TBI animals (67%) also showed subdural hematoma (SDH; Figure 1D).

**Figure 2:**
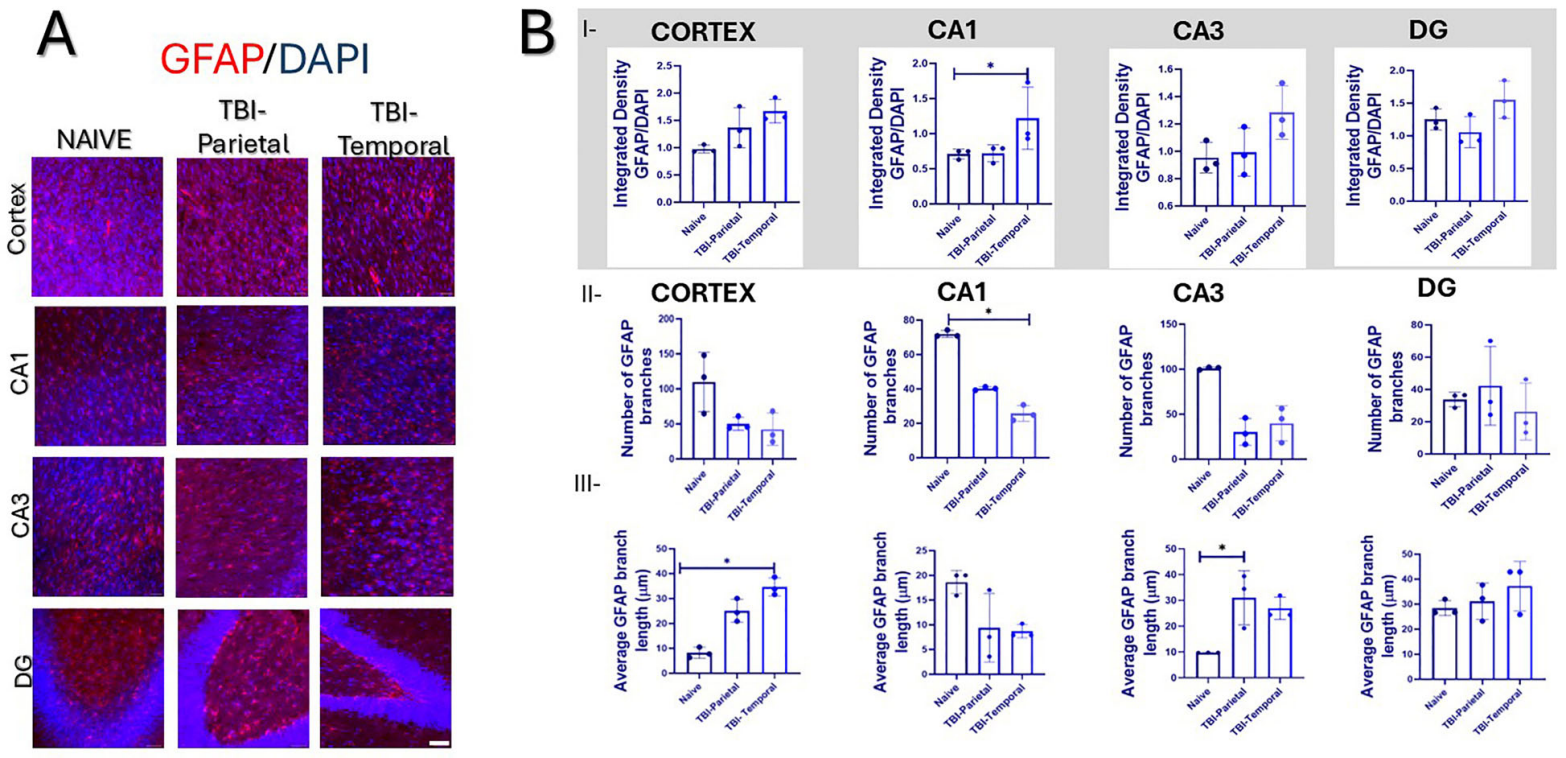
Astrocytic immunofluorescence in the cortex and hippocampus. **A,** Cortex and hippocampus photomicrographs of monoclonal mouse anti-glial fibrillary acidic protein (GFAP)-positive cells (in red) and nuclei (in blue, DAPI). **B**-**I**, Integrated density analysis of the entire image for GFAP per group. **B**-**II**, GFAP-positive branches. **B**-**III**, GFAP branch length analysis. Data are reported as means±SD. *P<0.05; Kruskal-Wallis test*;* n=9; three animals per group, each dot represents an animal. Scale bars=40 μm.

Histological analysis using cresyl violet staining revealed significant group differences in the lesion volume (Kruskal-Wallis: χ^2^=5.956, n=9, P=0.025), with the TBI-Temporal group showing larger lesions (0.098±0.035 mm^3^) than Naive (0.0027±0.0012 mm^3^; P=0.0171; values reflect baseline system sensitivity; Figure 1E I-II). Immunofluorescence analysis revealed a significant increase in GFAP integrated density when considering the entire image, specifically in the CA1 region (Kruskal-Wallis: χ^2^=5.69, n=9, P=0.036), with the TBI-Temporal group showing higher values (1.22±0.44) compared to Naive (0.71±0.69; P=0.0241; Figure 2B-I). In contrast, GFAP skeleton analysis indicated a significant reduction in the number of branches in CA1 (Kruskal-Wallis: χ^2^=6.00, n=9, P=0.0214), with the TBI-Temporal group displaying fewer branches (25.78±4.58) than Naive (72.00±2.02; P=0.0498; Figure 2B-II). Interestingly, GFAP average branch length was significantly greater in the cortex (Kruskal-Wallis: χ^2^=7.20, n=9, P=0.003), where the TBI-Temporal group exhibited longer GFAP branches (34.70±3.58 µm) than the Naive group (8.18±2.16 µm; P=0.0219). In CA3, there was a statistically significant difference among groups (Kruskal-Wallis: χ^2^=5.65, n=9, P=0.039), where TBI-Parietal groups had longer branches (31.03±10.52) than Naive (9.73±0.04; Figure 2B-III). Data of the GFAP analysis is shown in Table 1.

**Table 1:** Individual values of astrocytes and microglia immunofluorescence of the nine marmosets used for standardizing the lateral fluid percussion injury (LFPI).

Animal ID by group	Region	Int Den GFAP	Number of GFAP branches	Average length of GFAP branches (μm)	Int Den Iba-1	Number of Iba-1 branches	Average length of Iba-1 branches (μm)
Naive #1	Cortex	0.93±0.23	148.00±27.94	6.27±0.39	0.75±0.25	27±5.59	28.92±6.92
	CA1	0.76±0.15	70.83±60.63	20.00±16.44	1.14±0.50	40.83±5.71	29.85±10.10
	CA3	0.92±0.31	99.33±45.80	9.78±2.59	1.17±0.68	35.42±5.17	24.72±3.81
	DG	1.21±0.36	36.50±21.00	26.67±16.10	0.84±0.33	36.00±6.00	27.60±4.17
Naive #2	Cortex	0.75±0.36	64.83±25.64	10.53±1.78	0.75±0.59	71.00±5.97	16.40±5.97
	CA1	0.63±0.31	74.33±56.79	15.90±8.53	0.65±0.35	50.58±15.20	34.37±33.57
	CA3	0.87±0.34	102±61.14	9.70±2.70	0.64±0.39	43.83±11.03	15.97±2.46
	DG	1.13±0.83	28.67±21.28	31.96±16.69	1.03±0.53	34.33±35.83	32.69±17.01
Naive #3	Cortex	0.94±0.30	116.67±40.66	7.74±1.89	0.54±0.32	37.83±14.08	24.54±6.94
	CA1	0.75±0.20	70.83±60.59	19.98±16.41	0.61±0.28	39.17±13.39	25.87±13.41
	CA3	1.08±0.18	101.50±48.62	9.71±2.70	0.53±0.23	39.83±11.55	22.06±7.01
	DG	1.43±0.77	36.17±21.28	26.62±16.38	0.81±0.48	22.82±14.78	37.66±11.20
TBI-Parietal #1	Cortex	1.31±1.04	45.50±55.60	33.08±30.79	0.75±0.40	37.44±25.74	58.49±46.17
	CA1	0.79±0.38	39.22±47.85	36.15±17.93	1.20±0.29	43.22±14.03	43.15±7.46
	CA3	1.18±0.26	28.33±26.91	39.39±12.65	1.23±0.13	37.22±11.13	44.82±14.72
	DG	1.33±0.48	33.33±29.22	37.67±24.61	1.22±1.80	37.22±17.01	45.52±17.57
TBI-Parietal #2	Cortex	1.76±0.63	44.72±29.08	24.38±12.13	0.76±0.31	31.67±16.32	24.71±7.34
	CA1	0.79±0.25	24.78±14.36	20.44±9.43	0.81±0.34	39.00±17.95	25.42±8.49
	CA3	0.83±0.35	16.53±11.83	34.37±17.95	1.57±1.24	29.78±14.52	27.78±4.79
	DG	0.93±0.29	24.33±10.96	32.25±16.96	0.76±0.33	28.00±19.48	40.67±24.10
TBI-Parietal #3	Cortex	1.03±0.37	61±14.76	20.90±3.41	0.71±0.35	25.89±13.34	40.29±14.23
	CA1	0.58±0.09	44.22±11.95	23.67±4.71	0.74±0.24	27.83±6.80	32.35±11.78
	CA3	0.97±0.38	45.91±36.45	19.22±20.32	0.90±0.45	27.78±11.98	35.65±11.76
	DG	0.91±0.34	70.09±70.35	23.24±12.10	0.70±0.29	14.83±6.83	51.70±12.78
TBI-Temporal #1	Cortex	1.92±1.86	24.61±11.76	31.65±13.21	0.72±0.16	30.33±9.00	55.86±45.02
	CA1	1.01±0.29	24.80±68.29	7.89±1.18	0.96±0.34	23.00±6.45	24.49±18.74
	CA3	1.50±0.88	56.28±73.59	24.43±16.50	0.82±0.23	19.78±8.35	43.21±15.31
	DG	1.85±1.22	46.33±55.04	42.89±23.51	0.86±0.37	17.11±3.98	54.48±37.31
TBI-Temporal #2	Cortex	1.56±0.49	39.00±7.40	23.81±4.97	0.76±0.49	29.00±6.96	26.16±6.07
	CA1	0.93±0.40	21.78±6.65	10.25±9.79	0.82±0.16	25.44±11.30	40.60±11.71
	CA3	1.12±0.45	18.28±3.53	21.21±12.93	0.69±0.19	22.50±9.59	39.47±15.78
	DG	1.53±0.94	19.22±4.99	10.27±10.83	0.76±0.38	20.50±5.08	38.60±7.35
TBI-Temporal #3	Cortex	1.53±0.59	68.33±18.50	20.82±2.49	0.68±0.23	25.00±7.05	26.47±6.62
	CA1	1.73±0.91	30.73±10.33	19.68±8.88	0.58±0.28	17.11±12.03	64.70±73.98
	CA3	1.23±0.38	44.89±32.78	27.46±9.53	0.62±0.20	18.00±5.34	44.09±12.69
	DG	1.28±0.27	13.33±6.56	24.77±10.33	0.54±0.23	10.61±4.24	59.72±21.35

Data are reported as means±SD. Int Den: integrated density; GFAP: monoclonal mouse anti-glial fibrillary acidic protein; TBI: traumatic brain injury; CA1: cornu ammonis 1; CA3: cornu ammonis 3; DG: dentate gyrus.

**Figure 3:**
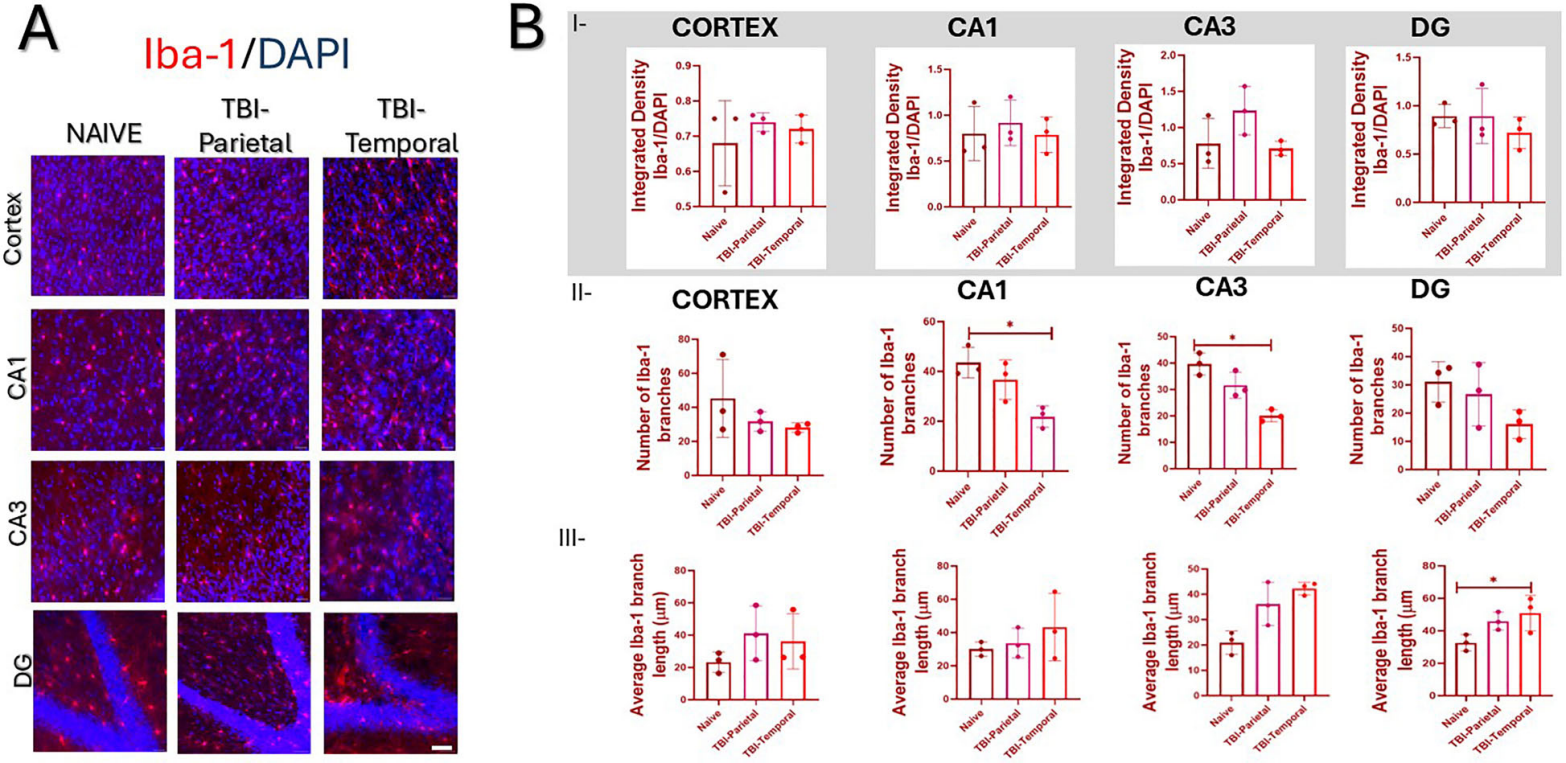
Microglial immunofluorescence in the cortex and hippocampus. **A**, Cortex and hippocampus photomicrographs of Iba-1-positive cells (in red) and nuclei (in blue, DAPI). **B**-**I**, Integrated density analysis of the entire image for Iba-1 for group. **B**-**II**, Number of branches of Iba-1-positive cells. **B**-**III**, Iba-1 branch length analysis. Data are reported as means±SD. *P<0.05; Kruskal-Wallis test; n=9; three animals per group, each dot represents an animal. Scale bars=40 μm.

Regarding microglia morphology, Iba-1 skeleton analysis indicated a significant reduction in the branch number in CA1 (Kruskal-Wallis: χ^2^=5.95, n=9, P=0.025), with fewer branches in TBI-Temporal (21.85±4.28, P=0.017) compared to Naive (43.53±6.16). Similarly, in CA3, a significant reduction in the Iba-1 branch number was observed (Kruskal-Wallis: χ^2^=6.48, n=9, P=0.0107), with fewer branches in the TBI-Temporal group (20.09±2.26, P=0.033) compared to the Naive group (39.63±4.21). Additionally, the Iba-1 average branch length was significantly increased in DG (Kruskal-Wallis: χ^2^=5.60, n=9, P=0.049), with longer branches in TBI-Temporal (50.93±11.00 μm, P=0.025) compared to Naive (32.65±5.03 μm; Figure 3B II-III). Data of the Iba-1 analysis is shown in Table 1.

**Figure 4:**
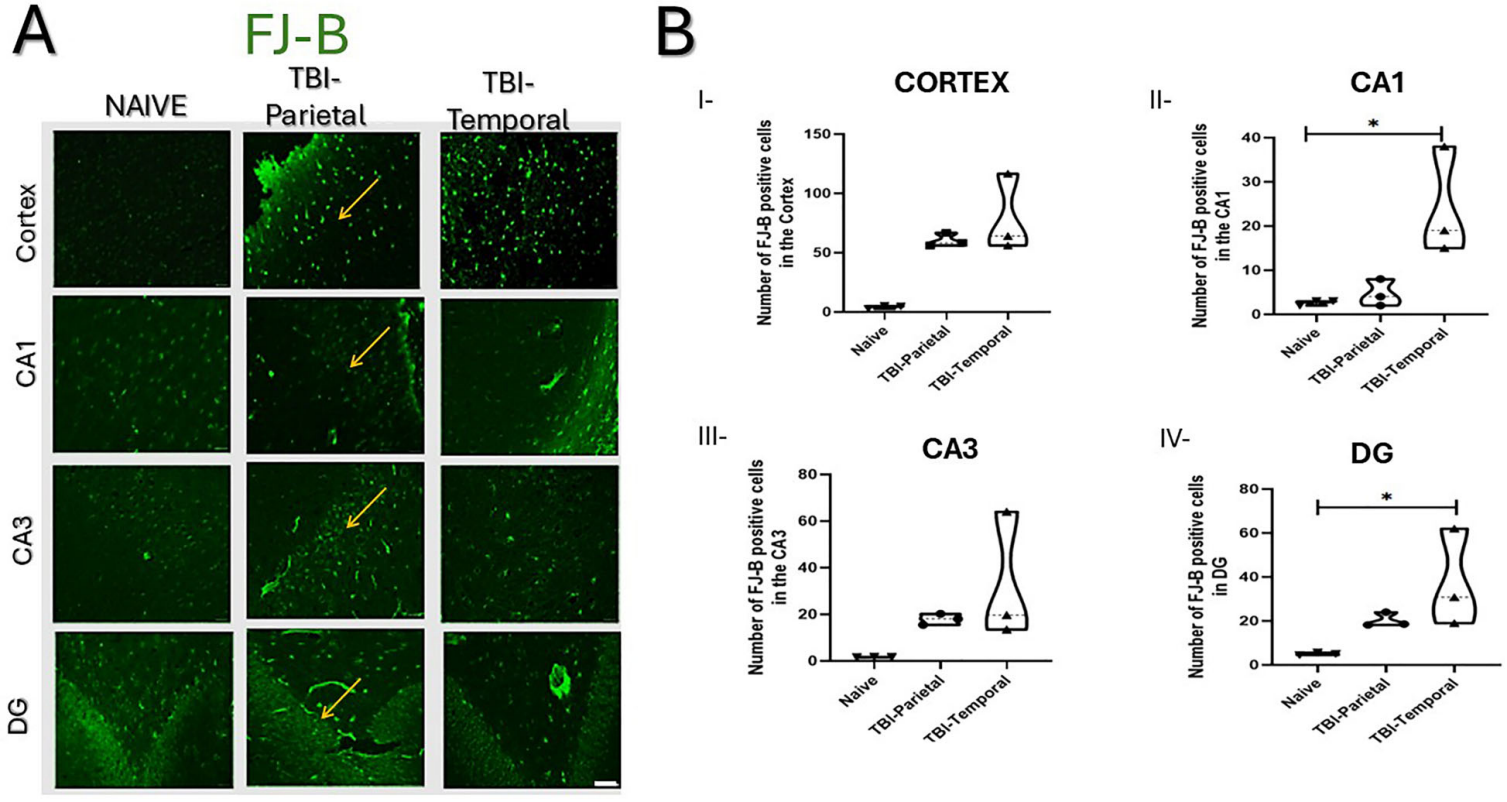
Fluoro Jade-B (FJ-B) staining in the cortex and hippocampus. **A**, FJ-B-positive cells in the cortex and hippocampus (arrows indicate positive cells). **B**, FJ-B quantification of degenerating neurons. **B**-**I**, Cortex; **B**-**II**, CA1; **B**-**III**, CA3; **B**-**IV**, DG. Data are reported as means±SD. *P<0.05; Kruskal-Wallis test; n=9; three animals per group, each dot represents an animal. Scale bars=40 μm.

Finally, neuronal degeneration assessed via FJ-B staining, revealed a significantly increased neuronal degeneration in both CA1 and DG regions (Kruskal-Wallis: CA1: χ^2^=5.85, n=9, P=0.025; DG: χ^2^=6.48, n=9, P=0.011; Figure 4A and B). Specifically, the TBI-Temporal group exhibited more degenerating neurons in CA1 (24.00±12.29 cells) and DG (37.27±22.22 cells) compared to the Naive group (CA1 2.66±0.57 cells, P=0.019; DG; 5.04±0.56 cells, P=0.033), highlighting the susceptibility of these hippocampal regions to trauma-induced damage.

## Discussion

To our knowledge, this is the first study to standardize the LFPI injury in common marmosets. Our primary aim was to identify the most suitable cortical region for inducing trauma and to explore the associated inflammatory response across different cortical areas.

In this study, the 17° angle was classified as moderate TBI because it consistently produced a longer righting reflex. Our choice aligned with clinical evidence, where prolonged unconsciousness is linked to more severe cognitive impairment, consistent with human TBI data (11). Notably, our results diverged from those reported in rodent models, where moderate TBI typically occurs at 2.4-2.8 atm and mild TBI at 1.8-2.2 atm (12). In rodents, mild TBI is defined by a 2-4 min righting reflex and 0-5% mortality; moderate TBI by a 6-10 min righting reflex and 10-20% mortality (13). In contrast, marmosets in our study exhibited more severe outcomes at lower pressures, suggesting that marmosets appear more sensitive to pressure-induced injury than rodents. These findings were consistent with our previous evidence of a greater sensitivity of marmosets in epilepsy models (14).

The occurrence of epidural (EDH) and subdural hematomas (SDH) in our marmosets subjected to TBI supports the clinical relevance of this species for modeling human TBI. Both EDH and SDH were extra-axial hemorrhages commonly observed in the clinical setting (15). While EDHs predict better outcomes, SDH is more commonly seen in moderate to severe TBI, linked to poor prognoses, as mentioned in clinical studies (15). Such hemorrhages are rarely reported in rodent LFPI models (16) despite evident vascular damage, underscoring a potential advantage of the marmoset model in better recapitulating human vascular responses to trauma, particularly in the temporal region.

Lesion volume analysis revealed significantly more extensive cortical damage in the temporal lobe compared to parietal injuries. This aligned with prior models in animal studies showing peak brain strain under the impact site (10-12%) (5) and highlighted the temporal lobe's vulnerability to compression and vascular injury. The observed lesion depths (range 0.8-0.9 mm) were consistent with prior reports on fluid-induced brain deformation in animal studies (5), reinforcing the validity of our model.

Regarding the astrocytic and microglial responses observed in marmosets, it is important to note that, although ferrets and swine have also been used as animal models to study TBI with the LFPI model - owing to their gyrencephalic brains, which better replicate the intracranial mechanical dynamics of humans compared to rodents (17-20) - the marmoset offers distinct advantages. Notably, its immune system and neuroinflammatory responses are more similar to those of humans (21).

Astrocytic response, assessed via GFAP staining, showed a marked reduction in the number of astrocytic branches in CA1 of the TBI-Temporal group. This finding may reflect the time-dependent nature of astrocyte proliferation reported in mice (22). Previous studies have shown that GFAP-positive astrocytes proliferate at 1, 3, and 7 days post-injury, with proliferating astrocytes peaking 3 days post-injury (22). This may explain the absence of significant changes in other regions. Notably, astrocytes near the lesion site appeared hypertrophic with elongated processes (22), similar to what we observed in marmosets.

Interestingly, the integrated density of GFAP in the CA1 region, as well as in other analyzed areas, was higher in the TBI-Temporal group compared to naive and TBI-Parietal animals. This pattern was consistent with findings from pig models of trauma that correlate with injury severity and have also been reported in human TBI, reinforcing the translational relevance of our observations (20). Supporting this interpretation, Lafrenaye et al. (23) demonstrated in micro pig models that serum GFAP levels measured 6 h post-injury were negatively correlated with the number of GFAP-positive astrocytes per cell, while positively correlating with GFAP integrated density. This correlation suggests that GFAP upregulation reflects astrocytic reactivity rather than astrocyte number alone.

Concerning microglia, our results suggest that TBI in the temporal lobe may induce greater inflammation than in the parietal lobe. The observed morphological changes, characterized by fewer but longer branches, indicate a reactive microglial phenotype, consistent with heightened inflammatory activity following temporal TBI (20). A reduction in the number of Iba-1-positive microglial branches in the hippocampus 24 h post-injury was also reported by Krieg et al. (24) in ferrets subjected to a different TBI model. Similarly, Lafrenaye et al. (19) demonstrated a reduction in the number of microglial processes following central fluid percussion injury in adult micro pigs. In contrast, Krieg et al. (24) observed an increase in Iba-1-positive microglia in the ferret cortex at the same time point, suggesting region-specific variation in microglial activation across species and injury types.

Moreover, it is important to highlight that in our study, flunixin meglumine, a nonsteroidal anti-inflammatory drug (NSAID), was administered as an analgesic after the trauma. Although some authors have reported that NSAIDs can attenuate microglial activation, flunixin meglumine has a short half-life of approximately 1.5 hours and is rapidly excreted in urine within 2-4 h after administration (25). Therefore, even if it had a transient effect on reducing microglial activation, any influence would likely have dissipated well before the 24-h post-trauma assessment.

Finally, the increased neuronal degeneration observed in the TBI-Temporal group compared to the Naive group further reinforces the temporal lobe's susceptibility. Limbic structures, including the hippocampus, are especially susceptible to hypoxic insults and excitotoxicity, and diffuse axonal injury commonly damages white matter tracts connected to the temporal lobe, such as the fornix (26). Although hippocampal degeneration was not significant across regions, the DG and CA1 showed more FJ-B-positive cells than CA3 (Figure 4B I-IV), possibly due to their proximity to ventricular fluid. The DG's molecular layer, rich in excitatory receptors and adjacent to the cerebrospinal fluid, may be more prone to glutamate accumulation and excitotoxicity following injury (27).

An important aspect to discuss in our marmoset neuronal degeneration data is how brain anatomy influences the distribution of mechanical forces during TBI. The lissencephalic brain causes stress to concentrate uniformly near the cortical surface (5). Although numerous rodent LFPI studies report significant hippocampal damage (2,28), this vulnerability is shaped by the relatively small brain size and the more superficial and superior orientation of the rodent hippocampus, which places it closer to the site of cortical impact (18). In contrast, in marmosets, the quasi-gyrencephalic (29) cortex allows forces to propagate deeper, as in other gyrencephalic species such as pigs, ferrets, and humans (3-5,18). This anatomical complexity led us to evaluate the effects of trauma across different cortical regions. Compared to rodents, marmosets exhibited greater sensitivity, with lower impact pressures resulting in more severe outcomes, including epidural and subdural hematomas and delayed righting reflex.

Moreover, temporal lobe injury produced more pronounced structural damage and inflammation than parietal injury, underscoring regional vulnerability and enhancing the translational value of the marmoset model for TBI research, in injury patterns that extend into deeper brain regions, including the sulci (3). Accordingly, the observed neuronal degeneration in the CA1 and DG of marmosets, which are regions distant from the cortical surface, highlights their greater translational relevance for modeling human TBI.

In conclusion, this study presents the first standardized application of the LFPI model in common marmosets, whereas other TBI models, such as cortical penetrating wounds, have been previously explored in this species (30). Here, we evaluated the effects of trauma across different cortical regions. Compared to rodents, marmosets exhibited greater sensitivity, with lower impact pressures resulting in more severe outcomes, including epidural and subdural hematomas and delayed righting reflex. Temporal lobe injury produced more pronounced structural damage and inflammation than parietal injury, underscoring regional vulnerability

Several limitations in our study should be acknowledged. The small sample size limits the statistical power of the findings; results should be interpreted cautiously and primarily serve to inform the design and power calculations of future, larger-scale studies. Moreover, the use of animals from a conservation facility, rather than captive-bred individuals, introduces uncertainty regarding potential prior trauma that may have influenced outcomes. Animal heterogeneity and analysis at a single time point also constrain interpretation, as TBI responses are dynamic and glial alterations and neurodegeneration evolve over days to weeks. This design captures only the acute phase, missing subacute and chronic effects. Furthermore, the absence of a sham group undergoing surgery without fluid impact limits the ability to distinguish injury-specific changes, particularly in glial responses, from those due to surgical or anesthetic stress. Ongoing studies with larger cohorts and suitable control groups are being performed to validate and extend these results.

## Data Availability

The datasets used and/or analyzed during the current study are available from the corresponding author upon reasonable request at the following link: https://docs.google.com/spreadsheets/d/1ngpeW_nCyBTGgTmUaawE7wHHJOXFq1NL-GcHBXfVAyk/edit?gid=395440289#gid=395440289.
